# The effect of an infection control guideline on the incidence of ventilator-associated pneumonia in patients admitted to the intensive care units

**DOI:** 10.1186/s12879-023-08151-w

**Published:** 2023-03-31

**Authors:** Ali Safavi, Shahram Molavynejad, Mahboobeh Rashidi, Marziyeh Asadizaker, Elham Maraghi

**Affiliations:** 1 Nursing Care Research Center in Chronic Diseases, School of Nursing and Midwifery, Ahvaz Jundishapur University of Medical Sciences, Ahvaz, Iran; 2 Pain Research Center, Ahvaz Jundishapur University of Medical Sciences, Ahvaz, Iran; 3 Department of Biostatistics and Epidemiology, Faculty of Public Health, Ahvaz Jundishapur University of Medical Sciences, Ahvaz, Iran

**Keywords:** Ventilator-Associated Pneumonia, Infection control guidelines, Nursing care

## Abstract

**Background and aim:**

Standard airway care can reduce the incidence of ventilator-associated pneumonia (VAP). This study aimed to determine the effect of implementing infection control guidelines on the incidence of VAP in patients admitted to the intensive care unit (ICU).

**Materials and Methods:**

In this clinical trial, 121 patients admitted to the intensive care units of Golestan and Imam Khomeini hospitals of Ahvaz, Iran who were under mechanical ventilation were assigned to two groups of control and intervention in non-randomly allocation. The study was conducted in two consecutive periods. In the intervention group, infection control guidelines were performed to prevent VAP and in the control group, routine care was performed. Data collection is done by used a three-part instrument. The first part included questions on the patients’ demographics and clinical information. The second part was the modified clinical pulmonary infection scale (MCPIS) for the early detection of VAP. The third part of the data collection instrument was a developed checklist through literature review. The MCPIS was completed for all patients on admission and the 5th day of the study.

**Results:**

The two groups were homogenous respecting their baseline characteristics (P > 0.05) including the mean MCPIS score (P > 0.05). However, the intervention group had lower body temperature (P < 0.001), lower white blood cell counts (P < 0.038), lower MCPIS score (P < 0.001), and higher PaO2/FIO2 (P < 0.013) at the end of the study. The incidence of VAP was significantly lower in the intervention group when compared to the control group (i.e. 30% vs. 65.6%, P < 0.001).

**Conclusions:**

The implementation of infection control guidelines could significantly reduce the incidence of VAP and its diagnostic indicators in patients admitted to the ICU. Nurses are advised to use these guidelines to prevent VAP in patients admitted to ICU.

## Introduction

It appears that “hospital-acquired infections” or “healthcare-associated infections” (HCAIs) can occur during the delivery of health care. Various studies in the United States and Europe have reported the incidence of HCAI to be between 13.0 and 20.3 cases per 1,000 patient-days. Invasive devices such as ventilators used in healthcare are associated with these infections [1].

Mechanical ventilators are commonly used in the intensive care units (ICU) to keep the patients alive [2]. However, patients under mechanical ventilation are exposed to a wide range of preventable pulmonary complications, including ventilator-associated pneumonia (VAP) [3]. VAP is defined as pneumonia occurred 48 to 72 h after endotracheal intubation and occurs in 9–27% of intubated patients [4]. In addition to affecting the patients’ outcomes, this infection increases health care costs both for patients and the healthcare system. Therefore, preventing VAP is a crucial issue in the management of patients admitted to the ICUs [5]. VAP is also associated with a significant increase in mortality, long-term use of intravenous antibiotics, increased dependence on mechanical ventilation, and prolongation of hospital and ICU stays [6, 7].

The risk of VAP increases by 5–65% per day in patients under mechanical ventilation [8].

Bacterial colonization of the oral cavity and aspiration of esophageal fluid play important roles in the pathogenesis of VAP. Some studies have also highlighted the role of gastric bacterial colonization and gastroesophageal aspiration in patients with a nasogastric tube and supine position in the pathogenesis of VAP [9]. Due to the high prevalence of VAP in ICUs and its high mortality, prevention is of particular importance and can help shorten the length of hospital stay and reduce healthcare costs [10].

Various strategies have been proposed to prevent VAP, including elevating the head of the bed and maintaining the tracheal cuff pressure between 20 and 30 cmH_2_O [10]. Prospective studies in patients admitted to ICUs have shown that keeping the head of the bed between 30 and 45 degrees could significantly reduce the risk of pulmonary aspiration and VAP [10, 11]. Some studies have also shown that patients whose endotracheal tube cuff pressure is less than 20 cmH_2_O are at higher risk of developing VAP. Therefore, maintaining the cuff pressure at 20–30 cmH_2_O can prevent aspiration of secretions accumulated in the subglottal region and the development of VAP [12, 13].

Bacterial colonization on the surface of healthcare workers’ hands is also one of the risk factors for transmission of infection and the development of VAP. Studies have also shown that good hand hygiene can reduce the risk of VAP [14], the length of stay in the ICU, and patient mortality [15]. A study also reported that hand hygiene, whether hand-washing or rubbing, can prevent VAP [16]. However, a study showed that despite the importance of hand hygiene in the prevention of VAP, only about 56% of ICU staff adhered to the hand hygiene protocols and this rate reached 65.5% after the educational intervention [17]. Currently, ICUs, like other departments of hospitals and health care centers, have infection control policies including washing hands in the main positions, observing the points of sterility and disinfection, how to use disposable devices, observing the hygiene of the environment and catheters, observing sterile techniques for intubation, suction and vein removal follow the notified programs and are sometimes updated. Therefore, conducting studies to evaluate the effectiveness of these guidelines is necessary. The primary objective of our study was to determine the effect of implementing infection control guidelines on the incidence of VAP. The secondary objective was to determine the effect of the implementation of the guidelines on the consumption of antibiotics in patients admitted to the intensive care unit.

## Methods

### Study design, setting, and definition

A clinical trial with a pretest-posttest design and not blinded was conducted after registration in the Iranian registry for clinical trials (registration number and date: IRCT20180709040402N1; 28.09.2018). The study was conducted on patients under mechanical ventilation admitted to ICUs of Golestan and Imam Khomeini hospitals in Ahvaz, Iran.

The sample size was calculated using the results of an earlier study [4] considering the type I and II errors at 0.01 and 0.2, and P1 and P2 at 0.3 and 0.1, respectively and the following formula, the minimum sample size was estimated at 59 per group. However, we recruited 65 patients in each group because of the potential attrition of 10%.

$$n = \frac{{{{({z_1} - a/2 + {z_\beta })}^2}[{p_1}(1 - {p_1}) + {p_2}(1 - {p_2})]}}{{{{({p_1} - {P_2})}^2}}}$$

Inclusion criteria included age over 18 years, having an orotracheal tube and being under mechanical ventilation, no ban for the elevation of the head of the bed, having no sign of pneumonia, cystic fibrosis, pleural empyema, neutropenia, and aspiration at the start of the study and till the first 48 h after the start of mechanical ventilation, and gaining a score < 5 from the modified clinical pulmonary infection scale (MCPIS).

Exclusions criteria were: Extrapulmonary infection, Patients with incomplete clinical information and records, Discharge of the patient from the special ward, transferring the patient from the ICU, return of consciousness and extubation of the patient and lack of ventilator dependence during the study, death of the patient.

The study was conducted in two consecutive periods. Patients admitted to the ICUs in the first period were allocated to the control group. After the sample size was completed in the control group, we recruited the patients in the intervention group. In each group patients with inclusion criteria were recruited consecutively. This method allowed researchers to prevent information contamination between the staff nurses involved in the two study phases. The control group was treated as usual, but those in the intervention group were treated according to VAP prevention guidelines. All participants or their legal guardians signed the written informed consent.

In the intervention group, the infection prevention guideline was implemented according to Table 2. Four of the main components of the guidelines are: (a) Observance of hand hygiene principles in five critical moments (i.e. before touching the patient, before aseptic procedures, after exposure to body fluids, after touching the patient, and after touching the patient’s surroundings), (b) frequent checking and adjusting the cuff pressure between 20 and 30 cmH_2_O (i.e. at 8 am, 4 pm, midnight, and after each oral care), and (c) keeping the head of bed elevate —between 30 and 45 degrees— and checking the right position using a goniometer three times a day (i.e. at 8 am, 4 pm, and midnight), and (d) frequent mouthwash using 0.2% or 0.12% chlorhexidine gluconate. A copy of the care guideline was installed over the beds of the patients in the intervention group to differentiate them from other patients in the ward.

**Table 1:** Modified clinical pulmonary infection scale (MCPIS)

Criteria	Score
**Temperature (°C)**	
36.5–38.4	0
38.5–38.9	1
< 36.0 or > 39.0	2
**Leukocyte count**	
4000–11,000	0
< 4000 or > 11,000	1
< 4000 or > 11,000 + over 500 bands	2
**Chest radiography**	
No infiltration	0
Diffuse or patchy infiltration	1
Localized infiltration	2
**Pulmonary secretions (present in the tracheal tube)**	
Absent	0
Present and non-purulent	1
Present and purulent	2
**PaO2/FIO2 (mm Hg)**	
> 240 or ARDS	0
≤ 240 and no evidence of ARDS	2

Patients in the control group received no intervention other than routine care. Routine care did not include frequent monitoring and adjustment of cuff pressure using a special device. Instead, the cuff pressure was traditionally estimated by the fingers and only after oral suction. The patient’s bed head remained raised, but this action did not follow a particular pattern and was sometimes flattened to prevent slipping of the patient. In addition, due to the nurses’ high workload and lack of time, hand hygiene was not strictly observed. Caregivers and type of intubation method were homogeneous in both groups.

The MCPIS was completed for all patients at their entry and then on the morning of the fifth day. All chest x-rays were interpreted by a radiologist and the laboratory tests were analyzed by the hospital laboratory. The body temperature of all patients was recorded axillary using a mercury thermometer for at least five minutes and then 0.5 °C was added to the reading [16, 17]. Chest physiotherapy was performed by a physiotherapist.

## Data collection

We used a three-part instrument to collect the study data. The first part included questions on the patients’ demographics and clinical information. The second part was the MCPIS. The MCPIS is a standard screening scale for the early detection of VAP. This scale includes five criteria: body temperature, leukocyte count, chest radiography, pulmonary secretion, and the PO2/FiO2 ratio. Each criterion is scored from zero to 2 as presented in Table 1. Then, the minimum and maximum scores can range between 5 and 10. Scores over 5 indicate VAP. The third part of the data collection instrument included a checklist (Table 2) we developed through literature review with focus the profile of Disease Control and Prevention Centers (CDC) and the American Thoracic Society for the Prevention of VAP [14, 18–25]. The checklist included items on the patient’s position, the type of the bed, use of the closed suction system, use of heat and moisture exchanger (HME), frequency of changing the suction catheters and equipment, air humidifiers, and ventilator tube set, frequency of chest physiotherapy, mouth care, change position, and suctioning of the subglottic secretions, frequency of cuff pressure monitoring and hand washing by the nursing staff, and deep vein thrombosis (DVT) and peptic ulcer prophylaxis.

**Table 2:** The checklist for evaluation of guideline-based interventions

Items	Explanations	Evaluation		Working shift
The patient is in a semi-recumbent position	-		Yes NO Yes NO Yes NO	Morning Evening Night
Using a standard ICU bed	-		Yes NO	*
Changing the suction catheters, connectors, and equipment	As required and for every new patient		Yes NO	*
Using a closed suction system	-		Yes NO	*
Using an orotracheal tube	-		Yes NO	*
Chest physiotherapy	At least once a day, by an expert		Yes NO Yes NO Yes NO	Morning Evening Night
Use of heat and moisture exchangers (HME)	Instead of humidifiers		Yes NO	*
Changing the HME	Weekly or as required		Yes NO	*
Suctioning the oral cavity and tracheal tube	As required		Yes NO	*
Observing the hand hygiene	In five critical moments (i.e. before touching the patient, before aseptic procedures, after exposure to body fluids, after touching the patient, and after touching the patient’s surroundings)		Yes NO Yes NO Yes NO	Morning Evening Night
Deep vein thrombosis (DVT) prophylaxis	According to the doctor’s order		Yes NO	*
Peptic ulcer prophylaxis	According to the doctor’s order		Yes NO	*
Mouthwash using 0.2% or 0.12% chlorhexidine gluconate	Every six hours		Yes NO Yes NO Yes NO	Morning Evening Night
Changing the patient’s position	Every two hours		Yes NO Yes NO Yes NO	Morning Evening Night
Monitoring and adjusting the cuff pressure between 20 and 30 cmH_2_O every eight hours and after each oral suction	Every eight hours		Yes NO Yes NO Yes NO	Morning Evening Night
Elevating the head of the bed —between 30–45 degrees— during and after gavages	During gavages		Yes NO Yes NO Yes NO	Morning Evening Night

The validity and reliability of the checklist were confirmed by 10 experts in intensive care including two anesthesiologists, two infectious disease specialists, two nursing instructors, two ICU nurses, and two infection control supervisors. We instructed the experts to use the AGREE II [26] international tool to assess the validity of the checklist.

### Statistical analysis

Data analysis was conducted using the SPSS software version 22. Quantitative variables were reported as mean, median, standard deviation, minimum and maximum, and qualitative variables were reported as frequency and percent. The normality of quantitative variables was assessed using the Shapiro-Wilk test. The chi-square or Fisher’s exact tests were used to compare the categorical and nominal variables between the two groups. The Mann-Whitney U test or t-test was also used for between-group comparisons of the quantitative variables according to the results of the normality test. The level of significance was set as < 0.05 in all tests.

## Results

### Patient characteristics and clinical data

**Fig. 1:**
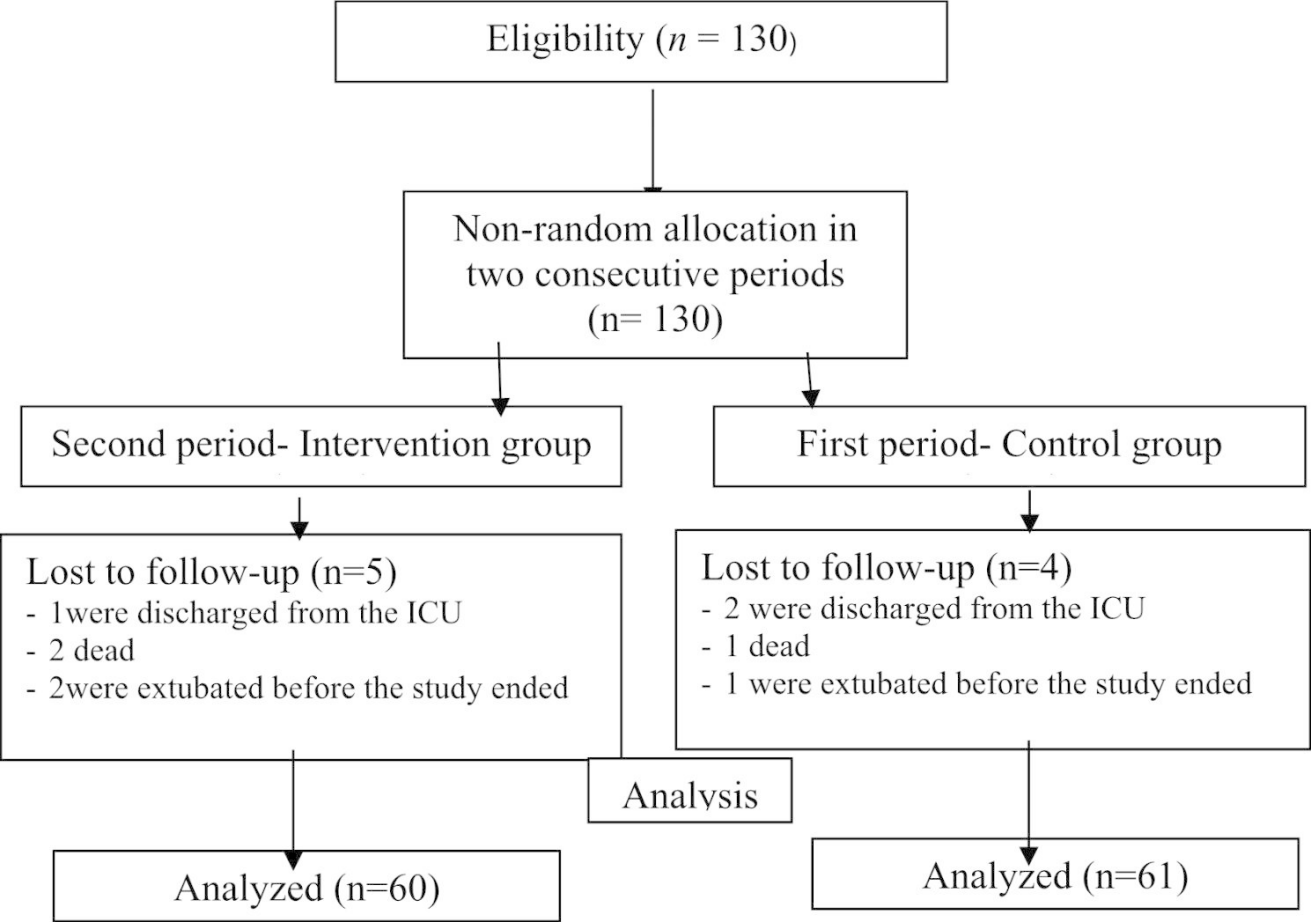
The study flow diagram

Out of 130 patients, 9 were excluded from the study (i.e., 4 from the control group and 5 from the intervention group) (Fig. 1). The mean age of the patients in the intervention and control groups was 43.48 ± 15.15 and 43.16 ± 15.44, respectively (P > 0.05). The two groups were homogenous respecting other baseline characteristics (P > 0.05) (Table 3), and underlying diseases (blood pressure, diabetes, diabetes-hypertension, kidney disease, cardiovascular disease, asthma, cancer, fatty liver and hyperlipidemia) (P = 0.621).

**Table 3:** Comparison of individual characteristics of intervention and control groups

Variables		Control group n (%)	Intervention group n (%)	Total	P value
Sex	Male	30 (49.2)	37 (61.7)	67 (55.4)	**0.167**
	Female	31 (50.8)	23 (38.3)	54 (44.6)	
Cause of hospitalization	Medical	31 (50.8)	28 (46.7)	59 (48.8)	**0.938**
	Surgical	10 (16.4)	9 (15)	19 (15.7)	
	Medical-surgical	12 (19.7)	14 (23.3)	26 (21.5)	
	Cardio-respiratory	8 (13.1)	9 (15)	17 (14)	
Having a comorbidity	Yes	29 (52.7)	26 (47.3)	55 (100)	**0.642**
	No	32 (48.5)	34 (51.5)	66 (100)	
Smoking	Yes	28 (45.9)	28 (46.7)	56 (46.3)	**0.933**
	No	33 (54.1)	32 (53.3)	65 (53.7)	

## Outcomes and estimation

Furthermore, we found no significant differences between the two groups respecting their baseline GCS, body temperature, white blood cell counts, Pao2/fio2, and MCPIS score (P > 0.05). However, the intervention group had lower body temperature (P < 0.001), lower white blood cell counts (P < 0.038), lower MCPIS score (P < 0.001), and higher PaO2/FIO2 (P < 0.013) at the end of the study (Table 4). Also, as presented in Table 5, the frequency of patchy and local infiltrations and purulent tracheal tube secretions were significantly lower among patients in the intervention group (P < 0.001). The incidence of VAP was significantly lower in the intervention group when compared to the control group (i.e., 30% vs. 65.6%, P < 0.001; Table 6). There was no significant difference between the two groups in terms of antibiotic use, peptic ulcer prophylaxis, and prophylaxis for deep vein thrombosis (P > 0.05).

**Table 4:** Comparison of baseline and post-intervention clinical characteristics of the intervention and control groups

Variables		Control group n (%)	Intervention group n (%)	P value
GCS	At entry	6.83 ± 1.0	6.93 ± 1.1	0.430
	On fifth day	6.95 ± 1.11	6.78 ± 1.0	0.270
Boy temperature	At entry	37.39 ± 0.89	37.56 ± 0.78	0.304
	On fifth day	38.48 ± 0.72	37.87 ± 0.84	0.001
White blood cell count	At entry	7483 ± 606.22	8336.0 ± 666.41	0.148
	On fifth day	11052.45 ± 435	9675.0 ± 451	0.038
PaO2/FIO2	At entry	215.26 ± 40.72	283.22 ± 55.32	0.513
	On fifth day	236.85 ± 54.57	256.59 ± 46.51	0.013
Total MCPIS score	Before the intervention	1.918 ± 1.158	2.167 ± 1.29	0.171
	On fifth day	5.541 ± 2.453	3.516 ± 2.534	0.001

**Table 5:** Post-intervention comparison of the Chest X-ray and tracheal tube secretions between the two groups

Variables	Group		P _Value_
	Control, n (%)	Intervention, n (%)	
**Chest x-ray**			0.001
With no change	14 (23)	36 (60)	
Patchy infiltration	24 (39.3)	19 (31.7)	
Local infiltration	23 (37.7)	5 (8.3)	
**Tracheal tube secretion**			0.001
No secretion	0	6 (10)	
None infective secretions	22 (36.1)	34 (56.7)	
Purulent secretions	39 (63.9)	20 (33.3)	

**Table 6:** Post-intervention comparison of the two groups in terms of VAP

VAP	Group		P value
	Intervention, n (%)	Control, n (%)	
Yes	18 (30)	40 (65.6)	0.001
No	42 (70)	21 (34.4)	

## Discussion

Our results confirmed the positive effects of nursing interventions based on the proposed guidelines on the incidence of VAP and its diagnostic indicators, with lower mean temperature and white blood cell count, and higher P/F ratios in the intervention group. The overall post-intervention mean MCPIS score was also lower in the intervention group, indicating the effectiveness of the intervention in the prevention of VAP. Yekefallah et al., in a study of investigate strategies for preventing VAP in ICU patients, have shown that effective suction of airway secretions and saliva reduces the incidence of VAP [27]. Bakhtiari et al. have also reported that a 5-day airway care program including cuff pressure adjustment, suctioning the subglottic secretions, and keeping the head of the bed at an angle of 45° could significantly reduce the mean MCPIS score [28]. Similarly, a study by Drakulovic et al. showed that raising the head of the bed can significantly reduce the incidence of VAP [29]. Another study also reported that a caring program consisting of frequent mouth care and airway suctioning, elevating the head of the bed, and hand washing before caring procedures could reduce the incidence of VAP [27]. Iwai et al. (2021) showed that sitting position exercise can significantly reduce the duration of mechanical ventilation [30].

Babaei et al. have also reported that a caring protocol including adjusting the cuff pressure at about 20 cmH2O, continuous suctioning of subglottic secretions, and keeping the head of the bed at 45° could keep the mean MCPIS lower in the intervention group than the patients who did not receive such cares [31]. Several earlier studies have also reported the same results when using similar care protocols. Our findings, together with those of previous studies [13, 14, 32], suggest that the use of infection prevention guidelines can significantly reduce the risk of VAP.

In the present study, the implementation of an infection prevention guideline could not significantly affect the use of antibiotics, peptic ulcer prophylaxis, and DVT prophylaxis. Previous studies have reported conflicting results in this regard. Righy et al. showed that the implementation of infection prevention guidelines did not affect the prophylactic use of antibiotics [33, 34]. However, Bouza et al. have reported that the implementation of infection control guidelines has reduced the use of antibiotics [35]. Due to the contradictory results of the studies and in support of Minozzi et al. (2021) proposal, and given the antimicrobial risk that occurs as a negative consequence of antibiotic use, further research with meticulous designs is needed to be conducted in this area [36].

## Conclusion

In the present study, the implementation of an infection control guideline could significantly reduce the incidence of VAP and its diagnostic indicators in patients admitted to the intensive care units. These findings confirm that designing and implementing simple and evidence-based guidelines can reduce the risk and incidence of VAP in the intensive care units. Then, the length of hospital stay and the costs will be reduced both for the patients and the healthcare system. This study has some limitations such as poor suctioning power of the central suction system, lack of tracheal tubes suitable for continuous subglottic suctioning, lack of real kinetic beds, and poor cooperation of some nurses due to overcrowding, made it difficult to conduct this study.

Due to the overuse of antibiotics in patients under mechanical ventilation, further studies are yet needed to examine the effect of implementing infection control guidelines on the use of antibiotics and other drugs in patients under mechanical ventilation.

## Data Availability

The datasets generated and analyzed during the current study are not publicly available to protect the participants’ confidentiality. However, they are available from the corresponding author on reasonable request.
